# Exploring the role of febuxostat’s drug target XOR in erectile dysfunction: insights from human genetics and rat models

**DOI:** 10.3389/fmed.2025.1674086

**Published:** 2025-11-21

**Authors:** Zhibin Chen, Yuqi Li, Chunyang Meng, Xiaorong Li, Huan Liao, Xiong Li, Yang Zeng, Xu Li, Tao Zhou, Qingfu Deng

**Affiliations:** 1 The Department of Urology, The Fisrt People’s Hospital of Neijiang, Neijiang, China; 2 Department of Urology, Affiliated Hospital of Southwest Medical University, Luzhou, Sichuan, China; 3 Department of Urology, Santai Hospital Affiliated to North Sichuan Medical College, Mianyang, China

**Keywords:** febuxostat, erectile dysfunction, apoptosis, Mendelian randomization, hyperuricemia-induced erectile dysfunction

## Abstract

**Background:**

The use of the uric acid-lowering drug Febuxostat (FB) has been associated with the risk of erectile dysfunction (ED) in men; however, findings from previous studies remain inconsistent. This study aimed to investigate the association between FB target genes and ED, as well as the underlying mechanisms involved.

**Methods:**

FB target genes were obtained from the DrugBank database. Mendelian randomization (MR) analysis was employed to determine the causal relationship between the target gene xanthine oxidoreductase (XOR) and ED. Molecular docking was then performed to assess the binding affinity between FB and XOR. A hyperuricemic rat model with ED was established, and several parameters were evaluated, including ICPmax/MAP ratio, serum testosterone, XOR, and p-eNOS/eNOS expression levels. In addition, levels of nitric oxide (NO), superoxide dismutase (SOD), malondialdehyde (MDA), and apoptosis in corpus cavernosum tissue were measured.

**Results:**

MR analysis revealed that XOR was significantly associated with an increased risk of ED (95% CI: 2.724–27.232; *p* < 0.001). Molecular docking confirmed a stable binding interaction between FB and XOR (binding energy: −8.2 kcal/mol). After 1 month of continuous oral administration of FB, XOR and MDA levels and the apoptosis rate in the corpus cavernosum were significantly reduced in hyperuricemic ED rats, while p-eNOS expression, the p-eNOS/eNOS ratio, and levels of NO and SOD were markedly increased.

**Conclusion:**

FB reducing oxidative stress and apoptosis in penile corpus cavernosum tissue in hyperuricemic rats by inhibiting XOR, thereby ameliorates ED.

## Introduction

Erectile dysfunction (ED) is defined as the inability to achieve or maintain a penile erection sufficient for satisfactory sexual intercourse (1). ED is influenced by various factors, including aging, metabolic disorders, neurological damage, and medication use. Among these, metabolic conditions such as diabetes mellitus, hypertension, hyperuricemia, and metabolic syndrome are significantly associated with the development of ED (2). With changes in lifestyle and dietary habits, the prevalence of ED related to metabolic dysfunction has markedly increased (3). It is estimated that by 2025, the global population affected by ED will reach 322 million (4). Phosphodiesterase type 5 inhibitors (PDE5is) are the first-line pharmacological treatment for ED; however, their efficacy is limited, with a response rate of only 60–70% (5). Therefore, there is a pressing need to explore novel therapeutic agents for ED.

Xanthine oxidoreductase (XOR) is a multifunctional enzyme that exists in two interconvertible forms: xanthine dehydrogenase (XDH) and xanthine oxidase (XO) (5). Both isoforms of XOR are involved in the terminal steps of purine metabolism, catalyzing the oxidation of hypoxanthine to xanthine and subsequently xanthine to uric acid (6). XDH exhibits dehydrogenase activity, transferring electrons from xanthine to NAD^+^ to form NADH. In contrast, XO exhibits oxidase activity, generating reactive oxygen species (ROS) such as superoxide anions (O₂^−^) during the oxidation process (7). Studies have shown that XOR expression is significantly upregulated in the corpus cavernosum tissue of ED rats compared to normal controls (8, 9), suggesting a potential role for XOR in the pathogenesis of ED.

Febuxostat (FB) is a potent XOR inhibitor that effectively reduces uric acid production (10). To date, only two studies have explored the relationship between FB and ED (11, 12). One study using a hyperuricemia-associated ED (HUED) rat model demonstrated that oral administration of FB significantly improved erectile function, indicating a potential therapeutic benefit for ED (12). Conversely, a 2022 propensity score-matched cohort study in U. S. patients with gout found that FB may increase the risk of ED compared to allopurinol in adult males. However, as noted by the authors, this study only compared the incidence of ED between the two treatment groups and did not clarify whether FB independently increases or decreases ED risk (11). Thus, the relationship between FB and ED remains inconclusive.

Mendelian randomization (MR) is a genetic epidemiological approach that uses single nucleotide polymorphisms (SNPs) as instrumental variables to assess the causal relationship between an exposure and an outcome. Because genetic variants are randomly assigned at conception and not influenced by environmental or lifestyle factors, MR can minimize confounding and reverse causation commonly present in observational studies.

**Figure 1:**
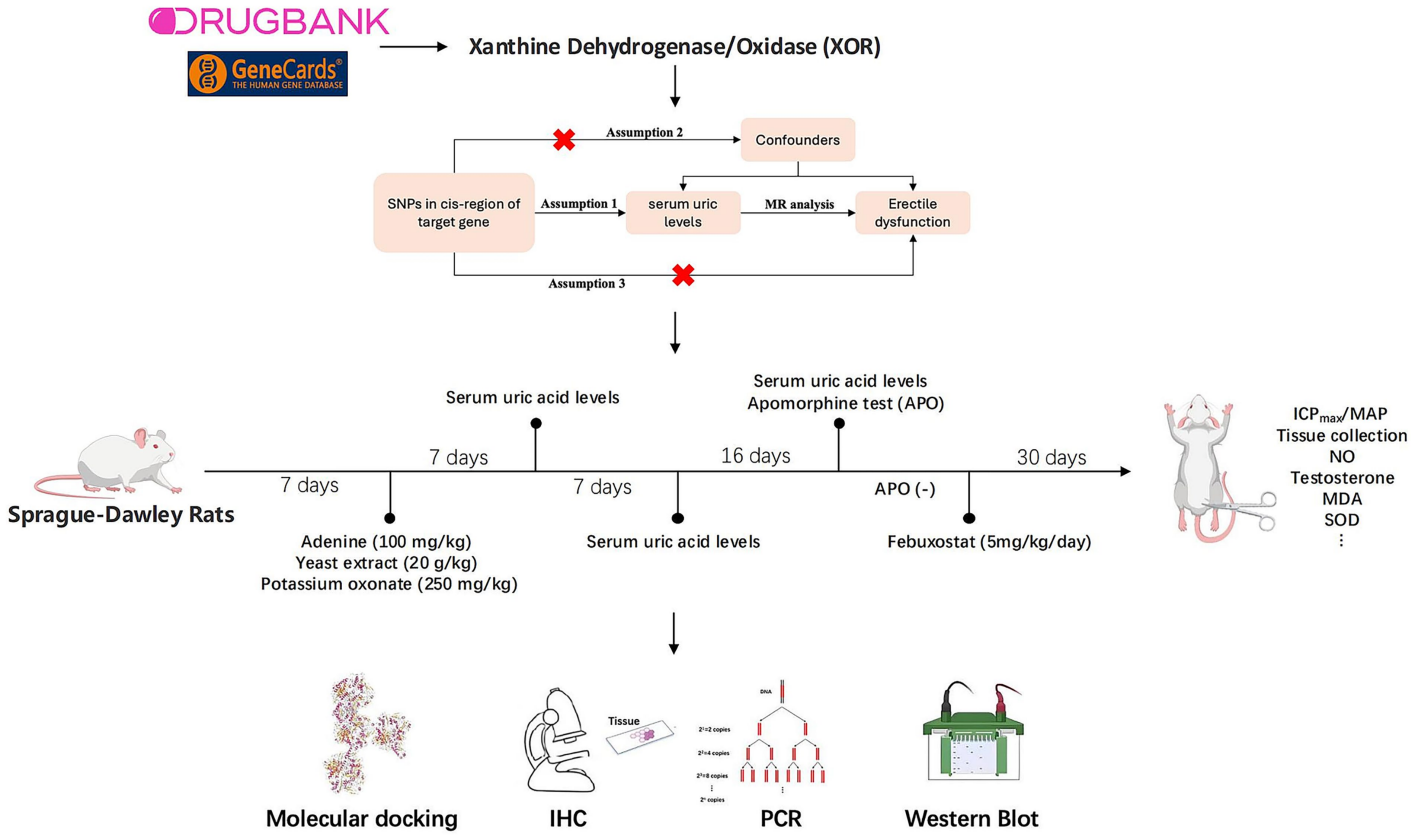
Study design overview.

In this study, we combined MR analysis with experimental validation using a HUED rat model to investigate the potential targets and mechanisms through which FB may improve erectile function. This is the first study to apply such a combined approach, providing novel evidence for drug repositioning and the identification of new therapeutic targets for ED. The detailed study design is illustrated in Figure 1.

## Materials and methods

### Molecular docking

We retrieved the target gene information of FB from the DrugBank^1^ and GeneCards^2^ online databases. The 3D structural file of FB was obtained from the PubChem database.^3^ To ensure accurate protein structures, we first searched for the UniProt Entry IDs of FB target genes via https://www.uniprot.org/, then downloaded the corresponding PDB-format protein files from the RCSB Protein Data Bank^4^ using these Entry IDs.

Molecular docking analysis was conducted using the CB-Dock2 online platform^5^ based on previously published methods (13, 14). Binding energy was used to evaluate the interaction between ligands and receptors: a binding energy < 0 kcal/mol indicates spontaneous binding, while a value < −5 kcal/mol indicates stable binding.

### Mendelian randomization

To ensure the validity of MR analysis, three core assumptions must be satisfied: (i) the selected SNPs are strongly associated with the exposure (relevance assumption), (ii) the SNPs are not associated with confounders of the exposure-outcome relationship (independence assumption), and (iii) the SNPs influence the outcome solely through the exposure (exclusion restriction assumption) (Figure 1) (15).

Instrumental variables within ±500 kb of the cis-region of FB target genes were selected from three genome-wide association studies (GWAS) related to serum uric acid levels. The selection criteria were *p* < 5 × 10^−8^ and *r*^2^ < 0.001. Two gout-related GWAS datasets were used as positive controls to validate the effectiveness of the selected IVs. All GWAS datasets included European populations to ensure consistency and reliability.

To reduce bias from weak instruments, we calculated the F-statistic for each SNP using the formula: *F* = *R*^2^ × (*N* − 2)/ (1 − *R*^2^), where *R*^2^ = β^2^ / (β^2^ + SE^2^ × (*N* − 1)) (16). SNPs with *F* > 10 were considered strong instruments and included in the subsequent analyses.

ED GWAS data were obtained from a study by Bovijn et al. (17), which included 223,805 European men from three cohorts, comprising 6,175 cases and 217,630 controls (18). Detailed dataset information is presented in Table 1.

**Table 1:** Summary of GWAS datasets used in this study.

Trait	Open GWAS ID	Sample size	PMID	Population	Statistical analysis
Serum uric acid levels	ebi-a-GCST90018977	343,836	34,594,039	European	Select IVs for drug targets in MR analysis
Urate levels	ebi-a-GCST90025965	437,354	34,226,706	European	
Urate levels (UKB data field 30,880)	ebi-a-GCST90014015	389,404	34,017,140	European	
Gout	ebi-a-GCST90038687	484,598	33,959,723	European	Positive control in MR analysis
Non-cancer illness code, self-reported: gout	ukb-b-13251	462,933	NA	European	
Erectile dysfunction	ebi-a-GCST006956	223,805	30,583,798	European	Outcomes in MR analysis

Details of all GWAS summary statistics used for instrumental variable selection, validation, and outcome analysis in the Mendelian randomization framework. NA, not available; UKB, UK Biobank; IVs, instrumental variables; MR, Mendelian randomization.

### Animal model and grouping

A total of 40 male SPF-grade Sprague–Dawley (SD) rats (8–10 weeks old) were purchased from the Experimental Animal Center of Southwest Medical University. After a 7-day acclimatization period, 30 rats were randomly selected to establish a hyperuricemic model. Based on previous protocols with modifications (19, 20), rats were administered adenine (100 mg/kg/day) and yeast extract (20 g/kg/day) by oral gavage, along with intraperitoneal injection of potassium oxonate (250 mg/kg/day) for 30 consecutive days. Blood samples were collected from the tail vein on days 7, 14, and 30 to measure serum uric acid levels. Rats with serum uric acid > 120 μmol/L at the final time point were considered successfully modeled.

Subsequently, rats with successfully induced hyperuricemia were administered subcutaneous apomorphine (100 μg/kg), and the presence of an erectile response within 30 min was observed to determine whether ED was present. Rats exhibiting an erectile response were classified as simple hyperuricemia model rats and excluded from subsequent experiments, whereas those without an erectile response were identified as HUED model rats.

A total of 24 HUED rats were randomly selected for further experiments. Based on different interventions, the animals were divided into four groups (*n* = 6 per group): normal control (NC), HUED, HUED + NaCl, and HUED + FB. Rats in the HUED + FB group received oral gavage of febuxostat (FB, 5 mg/kg/day) daily for 1 month, according to previous studies (12), while those in the HUED + NaCl group were administered an equivalent volume of normal saline by gavage for 1 month.

All procedures were approved by the Ethics Committee of the Affiliated Hospital of Southwest Medical University (Approval No. swmu20250063). All efforts were made to minimize animal suffering, and experiments were conducted in accordance with institutional guidelines for animal care.

### Measurement of serum uric acid

Serum uric acid levels were measured using a commercial assay kit (Biosharp, China). Blood (1 mL) was collected from the tail vein, allowed to clot, centrifuged, and the serum was analyzed according to the manufacturer’s instructions.

### ICP and MAP measurements

Rats were anesthetized with 5% isoflurane and maintained under 1.5% isoflurane during surgery. A tracheostomy was performed, and animals were ventilated. The corpus cavernosum and left common carotid artery were cannulated using heparinized 24G and 26G needles, respectively. Mean arterial pressure (MAP) and intracavernosal pressure (ICP) were recorded using pressure transducers connected to the BL-420S physiological recording system (Chengdu Techman, China). Electrical stimulation of the cavernous nerve was performed using the following parameters: 5 V, 12 Hz, 5 ms pulse width.

### Measurement of serum testosterone

Blood (2 mL) was collected from the common carotid artery and centrifuged to isolate serum. Free testosterone (F-TESTO) levels were measured using a commercial ELISA kit (Elabscience, China). Absorbance was measured at 450 nm using a microplate reader.

### Tissue NO, MDA, and SOD assays

After recording ICPmax and MAP, rats were euthanized by overdose anesthesia, and penile corpus cavernosum tissues were harvested. Tissues were homogenized and prepared for biochemical analysis. Commercial kits were used to measure nitric oxide (NO, Beyotime Biotechnology, China), malondialdehyde (MDA), and superoxide dismutase (SOD) levels (Nanjing Jiancheng Bioengineering Institute, China). Absorbance was read at corresponding wavelengths using a microplate reader.

### RNA extraction and PCR

Total RNA was extracted from corpus cavernosum tissue using an RNA extraction kit (FOREGENE, RE-03011, China), and RNA purity was confirmed via spectrophotometry (260/280 ratio). One microgram of RNA was reverse transcribed into cDNA using the ReverTra Ace qPCR RT Master Mix (Toyobo, Japan). PCR amplification was performed using XOR-specific primers (Forward: AACACAGCCTTCAGAGGTTT; Reverse: AGTCAGGTCCCCTTCTTTG) and SYBR Green reagents in a 10 μL reaction. Cycling was conducted on an ABI 7500 real-time PCR system (Thermo Scientific, United States) with the following conditions: 95 °C for 60 s, followed by 35 cycles of 95 °C for 15 s, 65 °C for 30 s, and 72 °C for 30 s. Gene expression was calculated using the ΔΔCT method.

### Immunohistochemistry

Rat penile corpus cavernosum tissues were sequentially fixed in paraformaldehyde, dehydrated, embedded in paraffin, and sectioned. After deparaffinization and rehydration, antigen retrieval was performed, followed by blocking of endogenous peroxidase activity and nonspecific binding sites. Tissue sections were incubated overnight at 4 °C with primary antibody against XOR (Proteintech, China, 1:50).

On the following day, the sections were incubated with HRP-conjugated goat anti-mouse secondary antibody (1:150, Easybio, China), followed by streptavidin–HRP incubation and visualization using diaminobenzidine (DAB) solution (Solarbio, China). Nuclei were counterstained with hematoxylin. Finally, the expression of XOR in the rat corpus cavernosum was observed under a light microscope.

### Total protein extraction and western blotting

Total protein was extracted from rat penile corpus cavernosum tissues using a total protein extraction kit (Solarbio, China). During the protein extraction process, 1% protease inhibitor and 1% phosphatase inhibitor were added to the lysis buffer to prevent protein degradation. All procedures were performed on ice to maintain a low temperature and minimize protein denaturation. Protein concentration was measured using the BCA protein assay kit (Thermo Scientific, United States) according to the manufacturer’s instructions to ensure equal protein loading among different experimental groups.

Protein samples were separated by SDS-PAGE under the following electrophoresis conditions: 60 V for 30 min followed by 120 V for 60 min to achieve optimal separation. Subsequently, the proteins were transferred onto a 0.45 μm polyvinylidene difluoride (PVDF) membrane using a constant current of 400 mA for 30 min. After transfer, the membrane was blocked with a rapid blocking buffer (Epizyme Biotech, China) for 15 min, followed by washing with Tris-buffered saline containing 0.1% Tween-20 (TBST) to remove unbound blocking agents. The membrane was then incubated overnight at 4 °C with appropriately diluted primary antibodies. The primary antibodies used included: XOR (Proteintech, China, 1:500), Bcl-2 (Proteintech, China, 1:5000), Bax (Proteintech, China, 1:5000), eNOS (Cell Signaling Technology, United States, 1:1000), p-eNOS (Cell Signaling Technology, United States, 1:1000), β-actin (Proteintech, China, 1:10000).

On the following day, the membrane was thoroughly washed with TBST to remove unbound primary antibodies. It was then incubated for 1 h at room temperature with horseradish peroxidase (HRP)-conjugated goat anti-rabbit or goat anti-mouse secondary antibodies (Easybio, China). After secondary antibody incubation, the membrane was washed again with TBST to reduce nonspecific binding. Protein bands were visualized using a chemiluminescence imaging system (Tanon, China), and band intensities were quantified using ImageJ software to determine the relative expression levels of the target proteins.

### Statistical analysis

Five MR methods were applied: The IVW method provides optimal statistical efficiency when all instrumental variables (IVs) satisfy the core assumptions, namely the absence of horizontal pleiotropy and effect heterogeneity. The MR-Egger method is suitable when horizontal pleiotropy exists among the instruments and can be used to detect and adjust for its influence on causal estimates. The weighted median method yields robust estimates when at least 50% of the weight comes from valid instruments, making it applicable in studies with uneven IV quality or the presence of some weak instruments. The simple mode method is appropriate when some IVs exhibit pleiotropy, but the majority are assumed to be valid. The weighted mode method is suitable when IVs may display heterogeneity and pleiotropy.

We assessed horizontal pleiotropy using MR-Egger regression and MR-PRESSO. Heterogeneity was evaluated using Q statistics from both IVW and MR-Egger. Leave-one-out analysis was conducted to assess the influence of individual SNPs on the exposure-outcome association, results were visualized using scatter plots, forest plots, and funnel plots.

All MR analyses were performed in R using the “TwoSampleMR” and “MR-PRESSO” packages. A two-sided *p* value < 0.05 was considered statistically significant.

Statistical analyses were performed using GraphPad Prism version 10 (GraphPad Software, United States). The Shapiro–Wilk test was used to assess the normality of the data. The Brown-Forsythe test or Bartlett’s test was used to evaluate the homogeneity of variances. Data were presented as mean ± standard deviation (M ± SD). One-way analysis of variance (ANOVA) was used for comparisons among multiple groups, followed by Tukey’s *post hoc* test. A *p*-value of < 0.05 was considered statistically significant.

Statistical analyses were performed using GraphPad Prism 10 (GraphPad Software, United States). Data are presented as mean ± standard deviation (M ± SD). The normality of all data was assessed using the Shapiro–Wilk test, and homogeneity of variance was evaluated using the Brown–Forsythe test. For parametric data that met the assumptions of normality and homogeneity of variance, one-way analysis of variance (ANOVA) followed by planned linear contrasts was conducted to test specific research hypotheses. Specifically, two contrasts were predefined: (1) HUED vs. NC, and (2) HUED+FB vs. HUED. For each outcome variable, only the *p* values for these two contrasts within the overall ANOVA model were reported, and all tests were two-tailed. When the assumption of homogeneity of variance was violated, Welch’s ANOVA combined with the Games–Howell *post hoc* test was used for verification. For nonparametric data that did not meet the assumptions of parametric tests, the Kruskal–Wallis test followed by Dunn’s post hoc test was applied, with Holm’s method used for multiple comparison correction for the two predefined contrasts. Unless otherwise specified, all p values were adjusted for multiple comparisons, and an adjusted *p* value < 0.05 was considered statistically significant.

## Results

### Target identification and molecular docking of FB

**Figure 2:**
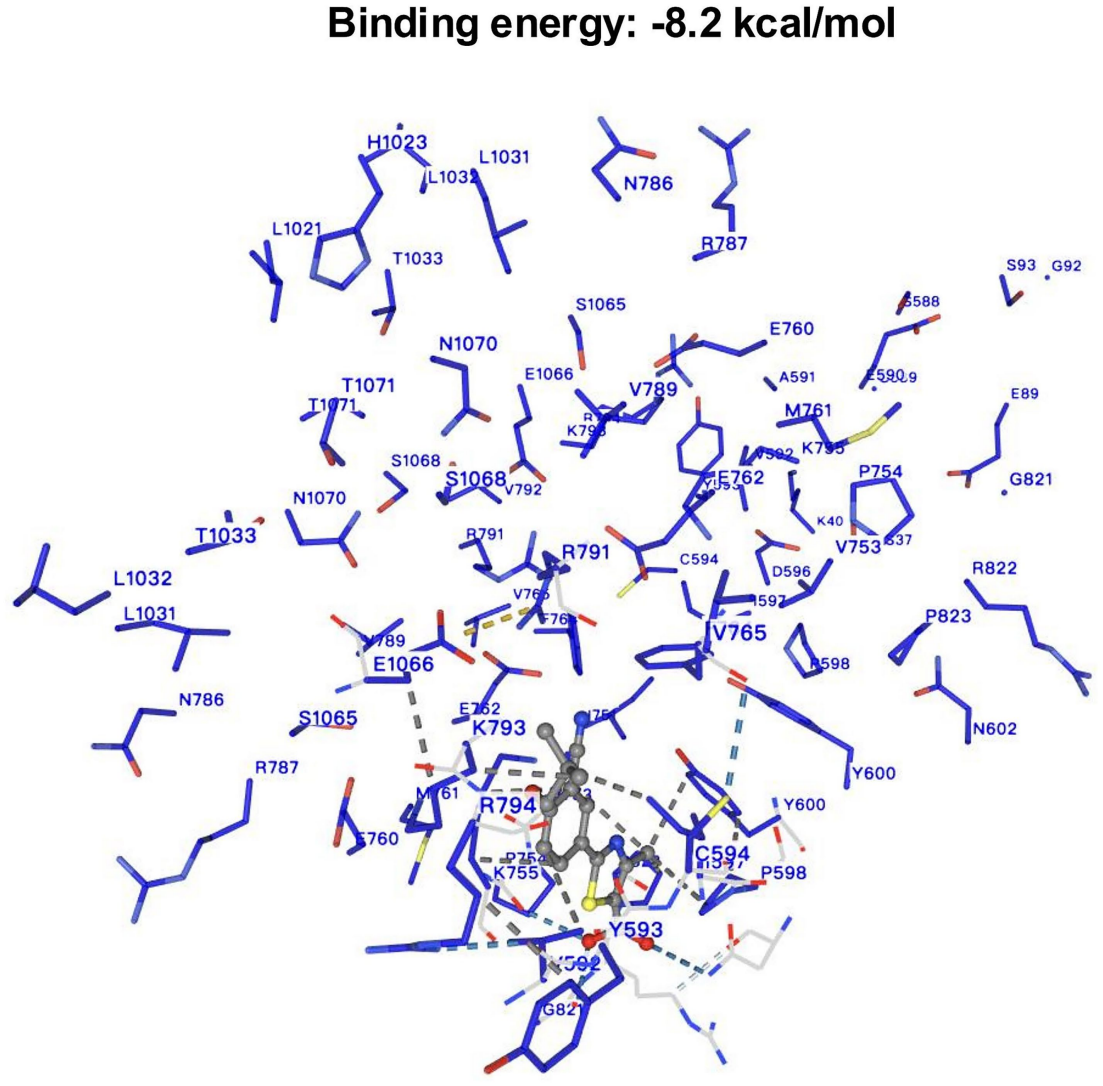
The result of molecular docking between febuxostat and XOR.

The DrugBank online database identified XOR as a target enzyme inhibited by FB. Subsequent molecular docking analysis showed a binding energy of −8.2 kcal/mol between FB and XOR, suggesting a stable interaction. As shown in Figure 2, FB binds primarily within the active pocket of XOR, involving several key residues, including charged residues (e.g., ARG31, ARG32, GLU89, LYS755, ARG791), hydrophobic residues (e.g., LEU36, VAL592, PHE764, ILE597), and polar residues (e.g., SER37, THR1033, ASN1070). Notably, residues such as ARG791 and LYS793 may stabilize the transition state, while aromatic residues like TYR593, TYR600, and PHE764 may enhance binding through *π*–π stacking or hydrophobic interactions. Additionally, acidic residues such as GLU762 and ASP595 may further optimize the binding through hydrogen bonds or salt bridges. These findings are consistent with previous studies, supporting that FB competitively occupies the XOR active site, thereby inhibiting substrate binding and suppressing uric acid production.

### Mendelian randomization analysis

**Figure 3:**
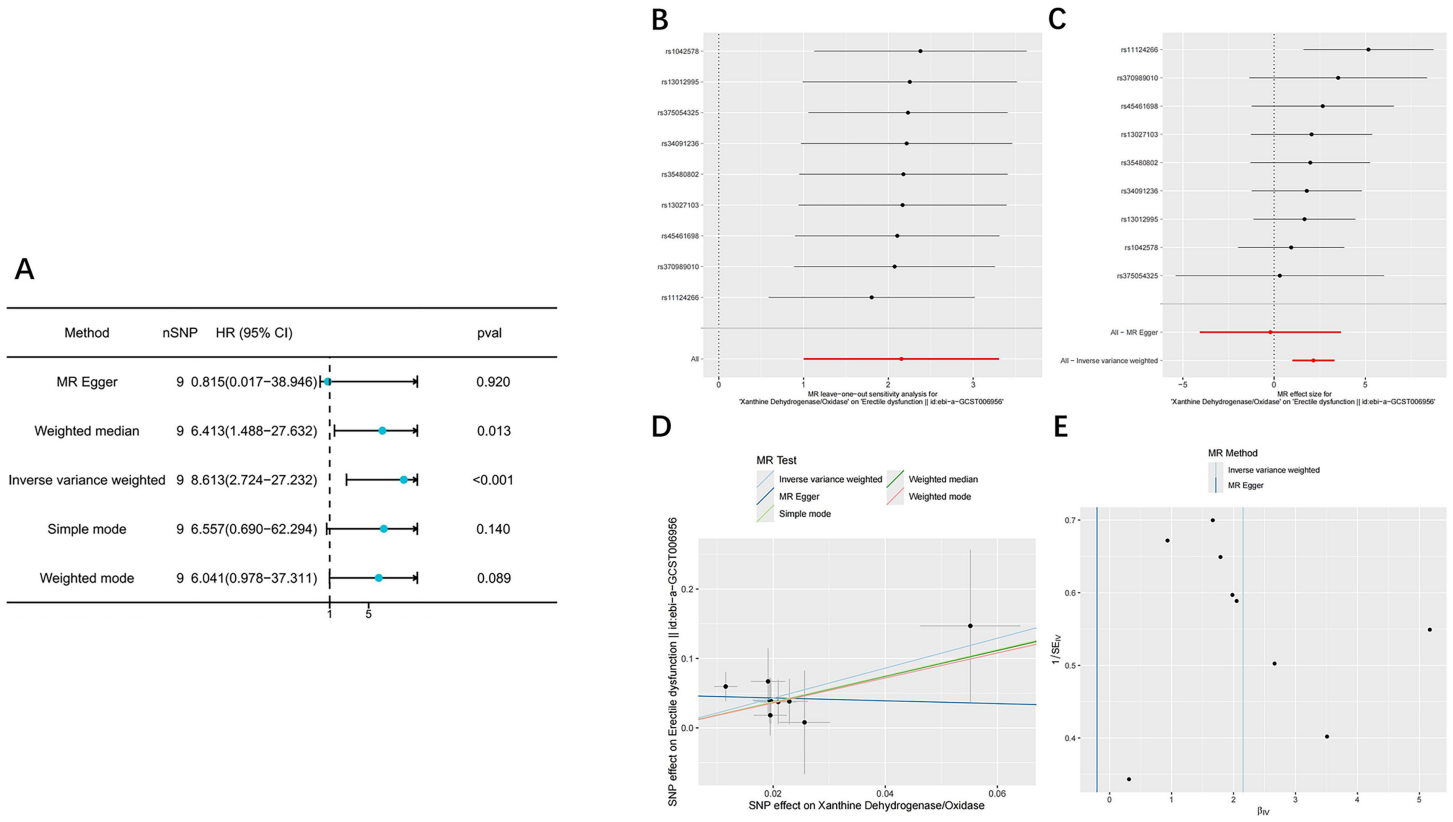
The result of Mendelian randomization analysis. **(A)** Forest plot showing causal estimates of ED obtained using five MR methods, including IVW, MR-Egger, weighted median, simple mode and weighted mode. **(B)** The Leave-One-Out plot of the effect of XOR on erectile dysfunction. **(C)** Forest plot displaying the individual causal effects of each SNP on ED, along with the overall IVW estimate. **(D)** The scatter plot of the effect of XOR on erectile dysfunction. **(E)** The funnel plot of the effect of XOR on erectile dysfunction.

A total of nine instrumental SNPs for XOR were identified in this study. All SNPs had F-statistics greater than 10 (Supplementary Table S1), indicating the absence of weak instrument bias. The final MR analysis demonstrated a significant positive causal relationship between XOR expression and the risk of ED (95% CI: 2.724–27.232; *p* < 0.001) (Figure 3A; Table 2).

**Table 2:** Summary of Mendelian randomization estimates using different analytical methods.

Method	nSNP	Beta	Se	Pval	OR	LCI95	UCI95	Q_pval	Mr_presso	Mr_pleiotropy
MR Egger	9	−0.205	1.973	0.920	0.815	0.017	38.946	0.903	0.851	0.251
Weighted median	9	1.858	0.745	0.013	6.413	1.488	27.632			
Inverse variance weighted	9	2.153	0.587	<0.001	8.613	2.724	27.232	0.823		
Simple mode	9	1.881	1.149	0.140	6.557	0.690	62.294			
Weighted mode	9	1.799	0.929	0.089	6.041	0.978	37.311			

MR analyses were performed using five different methods, MR Egger, weighted median, inverse variance weighted (IVW), simple mode, and weighted mode. nSNP, number of instrumental SNPs used; Beta, causal effect estimate; Se, standard error; Pval, *P*-value for the effect estimate; OR, odds ratio; LCI95/UCI95, 95% confidence interval lower and upper bounds; Q_pval, *p*-value from Cochran’s Q test for heterogeneity; Mr_presso, global test *p*-value assessing outlier effects; Mr_pleiotropy, MR Egger intercept *p*-value testing for directional pleiotropy. *P* < 0.05 was considered statistically significant.

Leave-one-out analysis (Figure 3B) showed that the overall causal estimates remained consistent after sequentially excluding each SNP, indicating strong robustness of the results. In the SNP-specific forest plot (Figure 3C), only one SNP exhibited a statistically significant causal effect, while the others were not significant; however, the overall IVW estimate remained statistically significant. This pattern suggests that the observed causal association is primarily driven by the collective effect of all instrumental variables rather than by any single SNP, thereby supporting the reliability of the overall causal inference. In the scatter plot (Figure 3D), the direction of the MR-Egger regression line was inconsistent with those of the other four methods, implying potential horizontal pleiotropy. The funnel plot (Figure 3E) displayed slight asymmetry, suggesting possible directional pleiotropy or minor small-sample bias. Notably, subsequent Cochran’s Q test, MR-PRESSO global test, and MR-Egger intercept test all yielded *p* > 0.05 (Table 2), indicating no significant heterogeneity or pleiotropy and supporting the robustness of the analysis results.

### Body weight, serum NO, uric acid, and testosterone levels in rats

Compared with the NC and HUED+FB groups, rats in the HUED and HUED+NaCl groups exhibited significantly elevated serum uric acid levels. In contrast, NO levels were markedly higher in the NC and HUED+FB groups than in the HUED and HUED+NaCl groups (Table 3). No significant differences in body weight or serum testosterone levels were observed among the four groups, suggesting that hyperuricemia induced ED is not dependent on changes in serum testosterone levels.

**Table 3:** Physiological and metabolic parameters in different groups (mean ± SD).

Group (*n* = 6)	Weight (g)	Serum uric levels (μmol/L)	ICP_max_ (mmHg)	MAP (mmHg)	NO (μmol/L)	Testosterone (nmol/L)	MDA (nmol/mgprot)	SOD (U/mgprot)
NC	526.00 ± 14.71	60.63 ± 30.56	82.16 ± 2.19	96.11 ± 7.19	12.8 ± 0.88	19.94 ± 0.62	1.85 ± 0.14	143.48 ± 9.39
HUED	527.83 ± 14.46	255.61 ± 16.22^#^	45.75 ± 4.94^#^	102.01 ± 7.39	4.83 ± 0.34^#^	20.00 ± 0.47	5.31 ± 0.61^#^	63.92 ± 5.88^#^
HUED+Nacl	522.00 ± 19.94	254.15 ± 10.32^#^	46.80 ± 3.98^#^	102.82 ± 6.88	4.92 ± 0.70^#^	20.04 ± 0.62	5.06 ± 0.39^#^	60.36 ± 3.41^#^
HUED+FB	518.00 ± 11.78	56.26 ± 13.45^*^	64.76 ± 1.18^*^	99.31 ± 7.16	12.15 ± 0.75^*^	19.97 ± 0.50	2.01 ± 0.43^*^	142.97 ± 8.06^*^

NC, normal control group; HUED, hyperuricemia model group; HUED+NaCl, hyperuricemic rats treated with normal saline; HUED+FB, hyperuricemic rats treated with febuxostat; ICP_max_, maximum intracavernosal pressure; MAP, mean arterial pressure; NO, nitric oxide; MDA, malondialdehyde; SOD, superoxide dismutase. ^#^*p* < 0.05, compared with NC; ^*^p < 0.05, compared with HUED.

### ICPmax/MAP ratio

**Figure 4:**
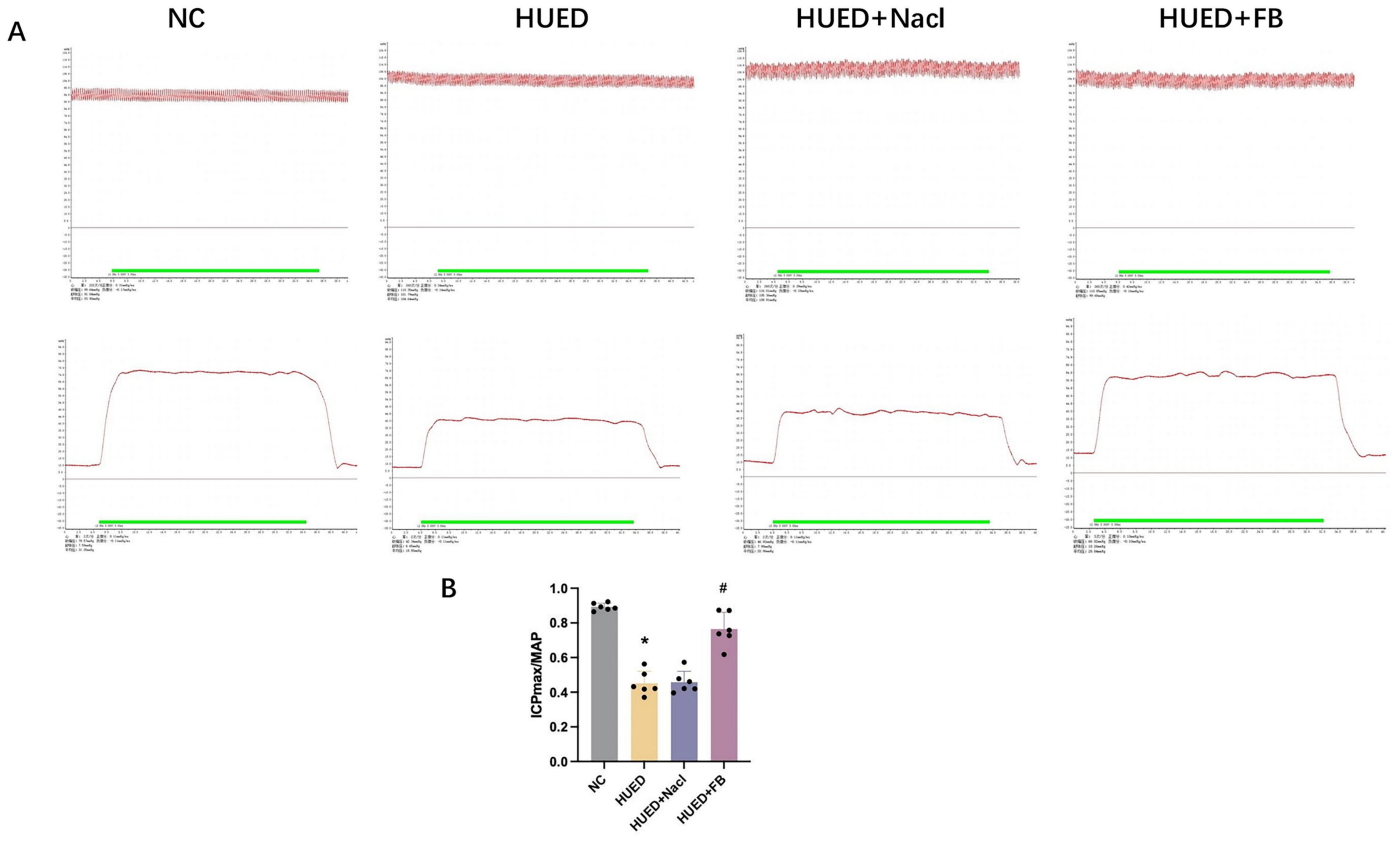
**(A)** Representative images of MAP and ICP_max_ in different groups under electrical stimulation at 5 V. **(B)** ICPmax/MAP in different groups. * Indicates *p* < 0.05 vs. NC group; # indicates *p* < 0.05 vs. HUED group.

The ICPmax/MAP ratio was significantly lower in the HUED and HUED+NaCl groups than in the NC group, while the HUED+FB group showed a significantly higher ratio than the HUED group. No significant difference was observed between the HUED+FB and NC groups (Figures 4A,B).

### MDA and SOD levels

MDA levels were significantly elevated in the HUED and HUED+NaCl groups compared with the NC and HUED+FB groups, whereas SOD levels were significantly reduced. No significant difference in MDA or SOD levels was observed between the NC and HUED+FB groups (Table 3).

### PCR

**Figure 5:**
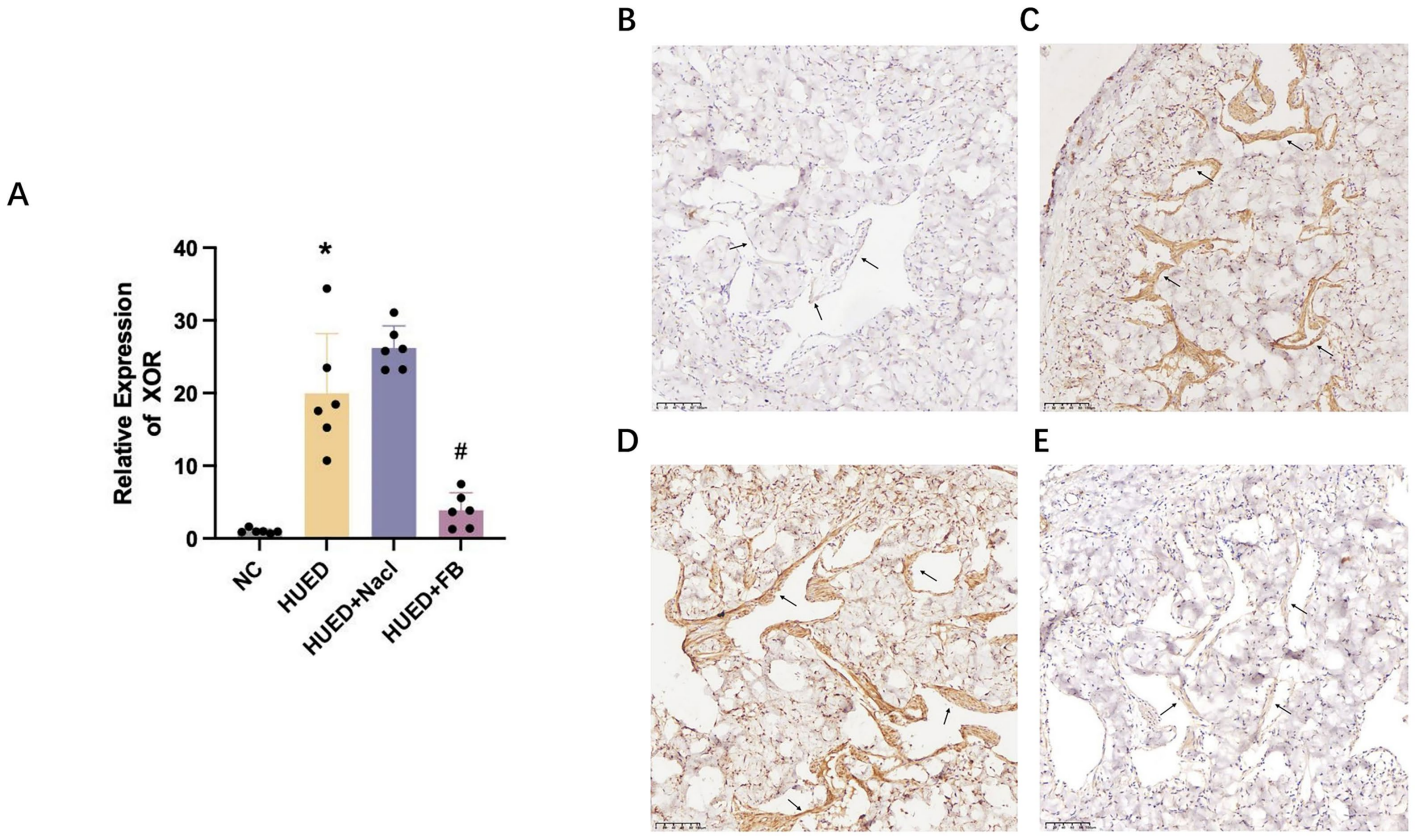
**(A)** mRNA expression levels of XOR in different groups measured by qRT-PCR. **(B)** Representative immunohistochemical staining of XOR expression in penile corpus cavernosum endothelium in NC group. **(C)** Representative immunohistochemical staining of XOR expression in penile corpus cavernosum endothelium in HU group. **(D)** Representative immunohistochemical staining of XOR expression in penile corpus cavernosum endothelium in HU + Nacl group. **(E)** Representative immunohistochemical staining of XOR expression in penile corpus cavernosum endothelium in HU + FB group. * Indicates *p* < 0.05 vs. NC group; # indicates *p* < 0.05 vs. HUED group.

PCR results indicated that XOR mRNA expression was significantly upregulated in the HUED and HUED+NaCl groups, whereas no significant difference was observed between the NC and HUED+FB groups (Figure 5A).

### Immunohistochemistry

Immunohistochemical analysis showed that XOR expression in the endothelial cells of the corpus cavernosum was markedly increased in the HUED and HUED+NaCl groups. In contrast, XOR expression in the HUED+FB group was comparable to that in the NC group (Figures 5B–E).

### Western blotting

In the corpus cavernosum of rats, XOR and BAX/BCL-2 protein expression levels were significantly higher in the HUED and HUED+NaCl groups compared with the NC and HUED+FB groups. Conversely, the p-eNOS expression and the p-eNOS/eNOS ratio were significantly lower in the HUED and HUED+NaCl groups. No significant differences in any protein expression levels were observed between the NC and HUED+FB groups (Figures 5C,D).

## Discussion

**Figure 6:**
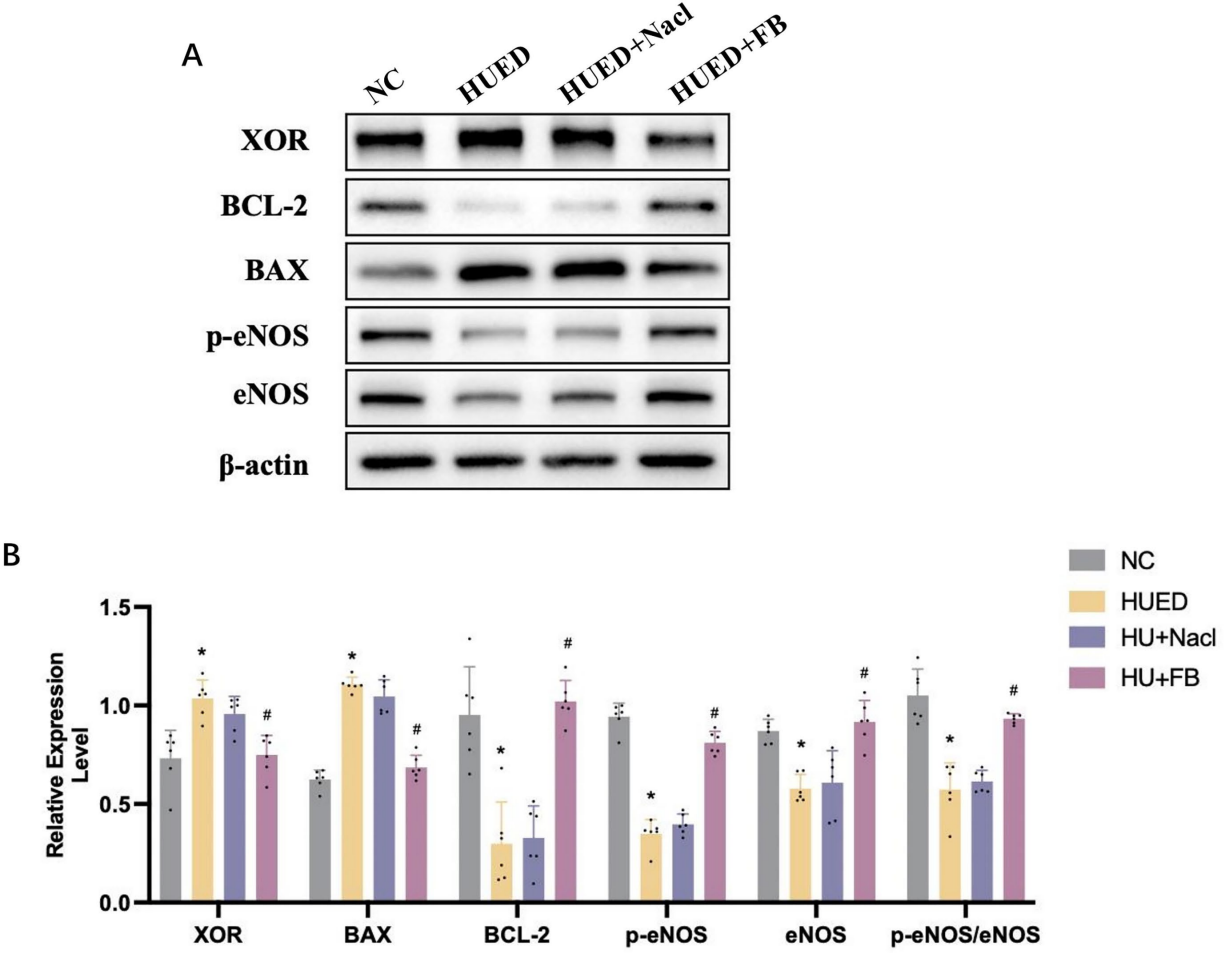
**(A)** Representative images of protein expression in different groups by western blotting. **(B)** Statistical analysis of protein expression in different groups by western blotting. * Indicates *p* < 0.05 vs. NC group; # indicates *p* < 0.05 vs. HUED group.

Although the development of novel therapeutic agents for ED continues to advance, phosphodiesterase type 5 inhibitors (PDE5i) remain the current first-line treatment, with an efficacy rate of only about 60–70% (21). In recent years, the rapid progress of multi-omics technologies has provided powerful tools for identifying new drug targets and biomarkers, thereby promoting both new drug development and target repurposing of existing drugs (21). Meanwhile, the integration of multiple analytical strategies facilitates the systematic elucidation of potential mechanisms linking drugs and diseases, offering valuable directions for scientists to explore disease pathogenesis and innovative therapeutic strategies (22) (Figure 6).

In this study, we combined MR analysis with animal model experiments to demonstrate the therapeutic efficacy of febuxostat in improving ED and to preliminarily explore its potential mechanisms. Febuxostat, a commonly used drug for hyperuricemia, has also been widely investigated for other diseases due to its remarkable anti-inflammatory properties (23–25). However, its relationship with ED remains unclear. Notably, while some population-based cohort studies have suggested that febuxostat may increase the risk of ED, animal studies have reported the opposite findings (11, 12, 26). This discrepancy may partly stem from species differences that lead to variations in pharmacokinetics and pharmacodynamics. Moreover, population studies often rely on electronic health record databases, which are susceptible to multiple biases such as data quality, selection bias, and comorbidities.

MR provides relatively robust causal evidence between genes and diseases at the genetic level in humans, whereas animal experiments offer direct mechanistic validation under controlled conditions. Our MR and molecular docking analyses indicated that elevated expression of the XOR gene significantly increases the risk of ED, while febuxostat may improve erectile function by binding to XOR with high affinity and suppressing its expression. Subsequent animal experiments further confirmed this mechanism: in HUED rats, the expression levels of XOR and apoptosis-related markers in penile corpus cavernosum tissue were markedly upregulated. In contrast, in the HUED + FB group receiving continuous oral febuxostat, the p-eNOS/eNOS ratio and NO levels were significantly elevated, while XOR expression and tissue apoptosis were notably reduced. These findings suggest that febuxostat may ameliorate ED by inhibiting XOR activity, thereby reducing apoptosis in penile corpus cavernosum tissue, activating the eNOS/NO signaling pathway, and ultimately improving erectile function.

XOR in its xanthine oxidase (XO) form transfers electrons from O₂ to monovalent or divalent states, producing superoxide anions (O₂•^−^) and hydrogen peroxide (H₂O₂). The xanthine dehydrogenase (XDH) form can also generate these reactive oxygen species (ROS) as a NADH oxidase (27). Under hyperuricemic conditions, electron transfer efficiency declines, favoring excessive ROS production (7). Excess ROS can reduce NO synthesis and bioavailability, leading to impaired erectile function (28). Additionally, chronic ROS overproduction diminishes antioxidant enzyme activity, increases oxidative stress, and ultimately causes endothelial injury (29). Malondialdehyde (MDA) and superoxide dismutase (SOD) are critical biomarkers of oxidative stress, with MDA positively and SOD negatively correlating with oxidative stress levels. In this study, MDA and SOD levels in the corpus cavernosum were measured to assess oxidative stress status. The HUED+FB group exhibited significantly lower MDA and higher SOD levels compared with the HUED and HUED+Nacl groups, along with reduced XOR expression, indicating that FB may mitigate oxidative stress by inhibiting XOR.

Apoptosis plays a key role in ED pathogenesis. Metabolic disorders and oxidative stress have been shown to directly trigger apoptosis (30). Accumulation of excess ROS also induces endothelial cell apoptosis, leading to decreased eNOS and NO levels, thereby impairing erectile function (31). As critical markers of apoptosis, changes in the expression of the pro-apoptotic protein BAX and the anti-apoptotic protein BCL-2 reflect the extent of apoptosis (32). In this study, the BAX/BCL-2 ratio was used to evaluate apoptosis in the corpus cavernosum. The BAX/BCL-2 ratio in the HUED+FB group was significantly lower than that in the HUED and HUED+Nacl groups, indicating that FB effectively inhibits apoptosis. In addition, several upstream regulatory factors, such as Nrf2, NF-κB, and PI3K/Akt, have been reported to participate in the regulation of XOR gene expression, as well as in modulating inflammatory responses and oxidative stress levels (33–35). These findings suggest that the beneficial effects of febuxostat on ED may also involve the participation of these regulatory pathways, which warrants further investigation in future studies.

It is worth noting that although our study demonstrated the beneficial effect of febuxostat on erectile function in patients with hyperuricemia, the safe dosing range and potential cardiovascular adverse effects of febuxostat have not yet been systematically evaluated. A multicenter clinical trial involving 6,190 patients reported that, compared with allopurinol, febuxostat treatment was associated with higher all-cause and cardiovascular mortality (36). However, another study enrolling 6,128 patients found that long-term febuxostat use did not increase the risk of death or serious adverse events (37). Furthermore, a real-world study from Taiwan involving 65,046 patients showed that febuxostat was associated with more favorable cardiovascular and cerebrovascular outcomes than allopurinol in individuals with hyperuricemia (38). Taken together, these findings indicate that the vascular safety of febuxostat remains uncertain. Therefore, when assessing its therapeutic efficacy for ED, it is essential to simultaneously define safe dosing parameters and monitor cardiovascular adverse events, while considering population characteristics, follow-up duration, and comorbidity burden to enable cautious and individualized clinical interpretation.

This study has several limitations. First, although MR provides causal inference at the genetic level, potential biases related to ethnicity and data quality remain unavoidable, and the results should be interpreted with caution. Second, this study evaluated only a single febuxostat dose (5 mg/kg/day) and a fixed treatment duration (1 month) in a hyperuricemia-related ED model, without systematically exploring dose–response relationships or treatment duration effects. Future studies should validate its efficacy in ED models with different etiologies and further determine the minimum effective dose, maximum tolerated dose, and exposure–response threshold. Finally, this study only preliminarily explored the potential febuxostat–apoptosis–ED mechanism. Future work should include comprehensive validation of upstream regulatory factors and downstream signaling pathways to strengthen mechanistic evidence.

## Conclusion

This study demonstrates that FB inhibiting apoptosis and oxidative stress in the penile corpus cavernosum, thereby ameliorates ED in hyperuricemic rats by targeting XOR. These findings provide new theoretical evidence for the development of precise molecular targets for ED therapy.

## Data Availability

The original contributions presented in the study are included in the article/Supplementary material, further inquiries can be directed to the corresponding authors.

## Footnotes

1
https://go.drugbank.com/

2
https://www.genecards.org/

3
https://pubchem.Ncbi.nlm.nih.gov/

4
https://www.rcsb.org/

5
https://cadd.Labshare.cn/cb-dock2
