# Supplementary material for: Exploring the role of febuxostat’s drug target XOR in erectile dysfunction: insights from human genetics and rat models
Source: Front Med (Lausanne). 2025 Nov 21;12:1674086. doi: 10.3389/fmed.2025.1674086 (PMC12678255; doi:10.3389/fmed.2025.1674086)
Supplement: Supplementary file 1 [file Table_1.docx]

| **Supplementary Table1.** **Summary of selected SNPs as instrumental variables for XOR in Mendelian randomization analysis** | | | | | | | | | | |
| --- | --- | --- | --- | --- | --- | --- | --- | --- | --- | --- |
| SNP | Chr | Pos | Beta | Se | Pval | Samplesize | Effect_allele | Other_allele | R2 | F |
| rs375054325 | 2 | 32086986 | -0.026 | 0.005 | 1.14E-08 | 343836 | G | A | 9.41E-05 | 32.360 |
| rs45461698 | 2 | 31570917 | -0.055 | 0.009 | 6.19E-10 | 389404 | C | T | 9.82E-05 | 38.258 |
| rs35480802 | 2 | 32010254 | -0.020 | 0.003 | 1.57E-10 | 389404 | A | C | 1.05E-04 | 40.941 |
| rs13027103 | 2 | 31891571 | -0.019 | 0.003 | 2.41E-10 | 389404 | A | G | 1.03E-04 | 40.099 |
| rs370989010 | 2 | 31751178 | -0.019 | 0.003 | 4.88E-10 | 389404 | T | A | 9.94E-05 | 38.721 |
| rs1042578 | 2 | 31749714 | -0.019 | 0.003 | 8.50E-12 | 437354 | T | C | 9.91E-05 | 43.359 |
| rs13012995 | 2 | 32007497 | -0.023 | 0.003 | 9.90E-13 | 437354 | T | C | 1.10E-04 | 47.948 |
| rs11124266 | 2 | 31918564 | 0.012 | 0.002 | 2.30E-08 | 437354 | C | A | 6.90E-05 | 30.185 |
| rs34091236 | 2 | 31901461 | -0.021 | 0.003 | 3.20E-11 | 437354 | T | C | 9.45E-05 | 41.336 |
| Basic characteristics of each selected SNP associated with XOR. SNP, single nucleotide polymorphism; Chr, chromosome number; Pos, base pair position (according to GRCh37/hg19); Beta, effect size of the association between the SNP and the exposure; Se, standard error of the effect estimate; Pval, P-value for the SNP–exposure association; Samplesize, total number of individuals included in the GWAS for the exposure; Effect_allele, allele associated with the exposure (used to calculate Beta); Other_allele, the alternative allele; R², proportion of variance in the exposure explained by each SNP; F, F-statistic, used to assess instrument strength (F > 10 indicates strong instruments). | | | | | | | | | | |
